# Myeloid-specific knockout of SHP2 regulates PI3K/PLCγ signaling pathway to protect against early myocardial infarction injury

**DOI:** 10.18632/aging.205096

**Published:** 2023-09-26

**Authors:** Menglin Sha, Hongxing Li, Bingyan Guo, Xiaoyong Geng

**Affiliations:** 1 Department of Cardiology, The Third Hospital of Hebei Medical University, Shijiazhuang, Hebei, China; 2 Department of Cardiology, The Second Hospital of Hebei Medical University, Shijiazhuang, Hebei, China

**Keywords:** SHP2, myocardial infarction, PI3K/PLCγ signaling pathway, endocytosis, inflammatory cytokine

## Abstract

Objectives: To study the effects of myeloid-specific knockout of SHP2 on early myocardial infarction and explore its molecular mechanism.

Methods: The model of myocardial infarction was established by using SHP2 in myeloid-specific knockout mice, and the effect of SHP2MAC-KO on myocardial function was detected by echocardiography. The effects of SHP2 on myocardial infarct size in myeloid-specific knockout mice was examined by TTC assay and Masson staining. Then, the detection of apoptosis was performed using TUNEL staining and inflammatory cell infiltration was observed using immunohistochemical staining. Moreover, macrophages in mouse hearts were selected by Flow Cytometry and treated with PI3K inhibitors respectively. Western blotting was then used to detect protein expression of p-SHP2 and PI3K/PLCγ signaling pathway. The phagocytic ability of cells was detected by endocytosis test, and the expression of inflammatory cytokines was detected by ELISA.

Results: Specific knockout of SHP2 in mice with myocardial infarction can improve the cardiac function, decrease infarct size, and reduce apoptosis as well as inflammatory cell infiltration. It also can mediate the PI3K/PLCγ signaling pathway in macrophages, which in turn enhances the endocytosis of macrophages and reduces the expression of inflammatory cytokines in macrophages.

Conclusions: Myeloid-specific knockout of SHP2 regulates PI3K/PLCγ signaling pathway to protect against early myocardial infarction injury.

## INTRODUCTION

Myocardial infarction (MI) is a type of myocardial necrosis caused by acute and chronic ischemia and hypoxia of the coronary artery, characterized by a sudden reduced blood flow to the myocardium, which ultimately leads to death from heart failure [1]. With cardiovascular disease accounting for more than one-third of global deaths so far, myocardial infarction is a pressing issue in today’s medical community [2]. Clinically, most first-line drug therapies for myocardial infarction focus on delaying the progression of the disease. Although they can delay the progression of myocardial infarction and reduce the mortality of patients, complications often occur in an unpredictable manner, including bleeding, myocardial ischemia-reperfusion injury and coronary restenosis [3–5]. Currently, the effect of the immunization therapy on myocardial infarction is remarkable. Therefore, exploring signaling pathways as potential therapeutic targets is crucial for the development of myocardial infarction therapy.

After myocardial infarction, the inflammatory response is enhanced in the injured myocardium [6, 7]. With ischemia and hypoxia, myocardial cells undergo apoptosis within hours to days. The damaged tissue triggers an inflammatory response which leads to the development of the granulation tissue. Immune cells infiltrate and release pro-inflammatory cytokines and chemokines, resulting in apoptosis [8]. It has been shown that PI3K activation can promote GRK2 S670 phosphorylation. Phosphorylation of GRK2 at residue S670 significantly impairs the interaction of GRK2 with the Gβγ subunit [9, 10]. The release of Gβγ eventually leads to the activation of phospholipase Cγ(PLCγ). Eukaryotic PI-PLC isozymes hydrolyze phosphatidylinositol 4,5-bisphosphate (PIP2) to produce DAG and inositol 1,4,5-triphosphate (IP3), a second messenger of calcification [11]. Mammalian pi-plcs include four subtypes, β, δ, γ, and ε [12]. All four subtypes contain a pleckstrin homology (PH) domain in their NH2-terminal region, allowing binding to specific phosphorylated phosphatidylinositol derivatives [13]. PLCγ isoenzymes can be regulated by tyrosine kinases in two ways. When cells are stimulated by growth factors that activate RTKs, PLCγ is recruited to the autophosphorylated tyrosine residues of RTK through its SH2 domain, resulting in phosphorylation of tyrosine and activation of PLCγ [14]. Studies have shown that Tyro3 family receptor tyrosine kinase (RTK)---MerTK, as a crucial protein in the process of clearing apoptotic cells, can be mediated by PLCγ2 [15]. PLCγ2 can prevent TIRAP being recruited to TLR4, and the MyD88-dependent signaling, indicating that PLCγ2 can inhibit the activation and production of MyD88 [16].

PTPN11, as a PTP family member, is a unique proto-oncogene [17], encoding Src-like adaptor protein 2-protein tyrosine phosphatase 2(SHP2), which is involved in multiple signaling pathways. SHP2 has long been considered an attractive target for the therapy of human disease. Therefore, it is speculated that SHP2 may regulate the injury of myocardial infarction by regulating PI3K/PLCγ signaling pathway.

## MATERIALS AND METHODS

### Construction of mouse model

Cell-specific SHP-2 deficient mice were screened from the offspring of Lyz2- cre+/-mice crossed with SHP-2folx/+ C57BL/6 mice using the cre-loxP gene targeting system. Myocardial infarction models were constructed on cell-specific SHP-2 deficient mice and wild-type mice [18, 19], which were purchased from the Center for Model Animal Research, Nanjing University (Nanjing, China). The construction methods were modified appropriately. Mice were anesthetized with 5% isoflurane, fixed in a supine position, and the left anterior thoracic surgical area was clipped and disinfected. A longitudinal incision of about 1.5cm in length was made at the skin about 2mm away from the left edge of sternum, and a vertical eversion mattress suture was performed at the incision to reserve suture. Next, we bluntly separated the muscles of chest wall layer by layer, quickly entered the chest cavity from the 4th intercostal space, opened the intercostal space with hemostatic forceps, and gently squeezed using left hand along with the heart beating to eject the heart from the intercostal space. Then, the heart was ligated with a suture needle through the anterior descending coronary artery at 2mm from the inferior edge of the left atrial appendage and 0.5mm from the conus pulmonalis. After the ligation, gently returned the heart to the chest cavity, squeezed the chest cavity to expel air and tightened the reserved suture at the ligation incision to complete the operation. Finally, the mice were fed with glucose saline after waking up. All mice were divided into four groups: sham: SHP2^WT^ group, sham: SHP2^MAC-KO^ group, Model: SHP2^WT^ group and Model: SHP2^MAC-KO^ group.

### Ultrasound detection of cardiac function in mice

After successful modeling and continuous culture for 3 days, the mice in each group were anesthetized with isoflurane, and their chest hair was removed. The cardiac structure after treatment was evaluated using Vevo 2100. Then, the two-dimensional echocardiographic results were obtained. The ejection fraction (EF) and shortening fraction (FS), left ventricular diastolic diameter (LVID; d) and left ventricular systolic diameter (LVID; s), systolic left anterior wall thickness (LVAW; s) and diastolic left anterior wall thickness (LVAW; d) can represent the cardiac structure and reflect the cardiac function.

### TTC

The hearts of mice in each group were continuously perfused with 3ml of 2% Evans Blue (Thermo Fisher, USA) from the aorta, placed neatly on an ice-cold iron plate, and stored overnight at -20° C. The frozen heart was removed and sectioned in a frozen state using a pre-cooled sharp blade evenly and serially along the long axis of the left ventricle to a thickness of 2mm per slice. The excised heart slabs were then placed in 2%TTC solution (Sigma Aldrich, USA) and incubated at 37° C for 15 minutes. After completion, myocardial sections were removed, rinsed with PBS, and fixed in 4% formaldehyde solution.

### TUNEL staining

According to the instructions of the TUNEL kit, the nuclei of TUNEL positive cells were stained brown under a microscope, while those of normal cells were stained blue. 5 fields of view were randomly selected to count TUNEL-positive cells and total cells under a fluorescence microscope, and apoptotic cells were counted with ImageJ software.

### Immunohistochemical staining

After the samples were removed and returned to room temperature, they were washed 3 times with PBS for 5 min each time. The samples were soaked fully with citrate buffer and repaired by low temperature heating in the microwave oven. After cooling, the samples were soaked in phosphate-buffered salt solution for 3 times with 5 min each time, and blocked for 20 min. The primary antibody was diluted to the target ratio with antibody diluent, then they were stored overnight at 4° C. After 30 min of rewarming, the samples were washed with PBS for three times with each time 5 min. Then, the secondary antibody was diluted with antibody diluent 3 times for 5 min each time and incubated for 2 h at room temperature away from light. Afterwards, the samples were eluted with PBS 3 times for 5 min each time, DAB staining, counterstaining, sealing.

### Masson staining

Mice were dissected, and heart tissues were collected, fixed with 4% paraformaldehyde, paraffin-embedded tissues, and 4μm thick sections were taken and stained with an affinity reagent. The sections were washed, stained with hematoxylin staining solution (Thermo Fisher, USA) for 3 minutes, and differentiated with hydrochloric acid-alcohol differentiation solution. After sections turned red, they were then stained with Masson acid staining solution with ponceau and acid fuchsin for 10 minutes, differentiated with 1% aqueous phosphomolybdic acid for 4 minutes, then stained with a light green solution for 5 minutes, and mounted in neutral resin. Finally, slides were taken under a microscope.

### Cell processing and grouping

The mouse leg bones in Model: SHP2^WT^ group and Model: SHP2^MAC-KO^ group were soaked into cold PBS, the ethanol on the surface of tibia and femur was washed away, the cleaned femur and tibia were separated, and the femur and tibia ends were cut with scissors. Then, the cold induction medium was aspirated with 1mL syringe to blow the bone marrow out of the femur and tibia, and repeatedly purged and washed 3 times until no obvious red color was visible in the leg bone. The medium containing bone marrow cells was repeatedly pipetted with a 5mL pipette to disperse the cell clumps, and then the cells were sieved using a 70μm cell filter, transferred to a 15mL centrifuge tube, centrifuged at 1500 rpm/min for 5 min. The supernatant was discarded, red blood cell lysate was added to resuspend for 5 min and centrifuged at 1500 rpm/min for 5 min. Then, the supernatant was discarded again and resuspended with a cold configured bone marrow macrophage induction medium and plated. The medium didn’t change during cell culture, half of the bone marrow macrophage induction medium was replaced after the third day of culture, all the medium was replaced on the fifth day, and it can be used for subsequent experiments on the seventh day. A portion of the macrophages were treated with the PI3K inhibitor 740Y-P (HY-P0175, MedChemExpress, USA), and cells were divided into four groups: control, KO, control + PI3K inhibitor, and KO + PI3K inhibitor.

### Phagocytosis assay with fluorescent microspheres

The macrophages in four groups were incubated with 10μm fluorescent silver YG microsphere latex beads, which was pre-incubated in 2.1% BSA (Polysciences, USA; 4.55x106 beads per ml) for 24 hours. The coverslips with cells were then washed extensively in PBS, fixed with 2% paraformaldehyde, counterstained with 4’,6-diamidino-2-phenylindole (for nuclei) and 1,1’-octacosanyl-3,3,3’,3’- tetramethylindocyanine (for membranes) according to the manufacturer’s instructions (Molecular Probes, USA), inverted and mounted on slides with VectorShield mounting medium (Vector Laboratories, USA). Digital images were captured by fluorescence microscopy using different excitation wavelengths of latex beads, 4’,6-diamidino-2-phenylindole, and 1,1’-octacosyl-3,3,3’,3’-tetramethylindoline. To quantitatively determine the uptake of latex beads by macrophages, the number of beads and nuclei was counted in the marked regions under each experimental condition.

### ELISA

The ELISA plate was coated with TNFα and IL-1β antigen, and 100μl antigen was added to each well, then incubated at 37° C for 4 hours and sealed with 5% calf serum at 37° C for 40 minutes. The diluted sample was added to enzyme-labeled reaction wells with 100μl per well, placed at 37° C, then the fluorophore-labeled antibodies (Abcam, ab150077) were added, kept at 37° C for 60 minutes. After washing, the 3,3’,5,5’-tetramethylbenzidine (TMB) was added, and the reaction was finally terminated. The value of OD was measured at 450nm wavelength with a microplate reader, and the concentration of TNFα and IL-1β were calculated by drawing a standard curve.

### Western blotting

5ul of phenylmethanesulfonyl fluoride (PMSF) and 5ul of phosphatase inhibitor cocktail were added to 490ul RIPA lysate to extract cell protein, and the sample protein concentration was measured by using BCA protein quantification kit. Then the gels for separation and concentration of protein should be prepared. The 5×SDS-PAGE Loading Buffer was added to the protein sample to dilute it into the sample containing 1×SDS-PAGE Loading Buffer. 30ug of protein sample was added through the well, and the maker was added into the first well on both sides, and the difference in sample volume can be made up with 1×SDS loading buffer. After switching on the power, 80V, 30 minutes to reach the interface of gels for separation, then set the voltage to 120V, 1.5 hours to reach the bottom of the gels for separation to stop electrophoresis. Then the membrane was transferred, and put in the TBST buffer containing 5% skim milk powder, blocked for 2 h. Primary antibodies p-SHP2(Abcam, ab62322, 1:1000), p-PI3K (CellSignaling, 17366, 1:1000), p-MerTK (CellSignaling, 44463,1:1000), p-PLCγ2 (CellSignaling, 3871, 1:1000), p-Myd88 (CellSignaling, 17366, 1:1000), p-NF-κB (CellSignaling, 3303,1:1000), TNFα (CellSignaling, 11948, 1:1000), IFNγ (CellSignaling, 8455, 1:1000), IL-1β (CellSignaling, 12703, 1:1000), and GADPH (CellSignaling, 5174, 1:1000) were added, and were incubated at room temperature for 1 hour, then incubated at 4° C overnight. After washing 3 times with TBST for 5 min each, the secondary anti-goat anti-rabbit IgG H&L (Abcam, ab6702, 1:5000) was added and incubated for 2 h, then washed 3 times with TBST for 5 min each. The color was exposed and developed, and then the result band was analyzed.

### Statistical analysis

GraphPad Prism 9 was used for data analysis and processing. The measured data was expressed as mean ± standard deviation (SD), and chi square test was used to analyze the comparisons among multiple groups, while t-test was used to analyze the comparisons between two groups. P<0.05 was considered statistically significant.

## RESULTS

### SHP2^MAC-KO^ could improve cardiac function and alleviate myocardial injury in mice with myocardial infarction

**Figure 1:**
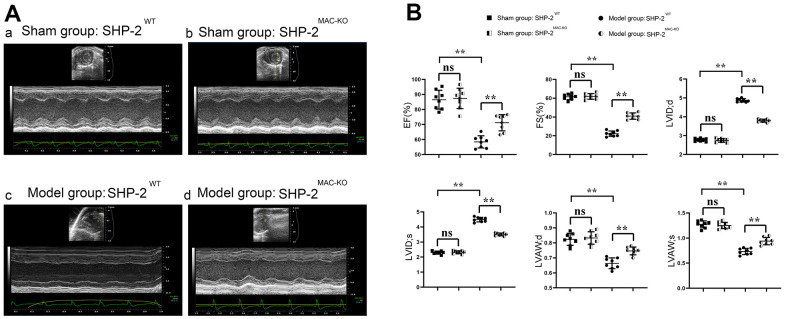
**SHP2^MAC-KO^ improves the cardiac function in mice with myocardial infarction.** (**A**) Ultrasound imaging of mice in sham: SHP2WT group, sham: SHP2MAC-KO group, Model: SHP2WT group and Model: SHP2MAC-KO mice; (**B**) Statistics of EF, FS, LVAW; d, LVAW; s, LVID; d and LVID; s. P<0.01, ns P>0.05.

The cardiac function of mice in each group was evaluated by ultrasound equipment. The results showed that compared with sham: SHP2^WT^, the expression of EF, FS, LVAW;d, LVAW; s, LVID; d and LVID; s in the group of sham: SHP2^MAC-KO^ had no significant difference. Compared with the group of Model: SHP2^WT^ group, the expression of EF, FS, LVAW; d and LVAW; s increased significantly, and the expression of LVID; d and LVID; s was significantly reduced in the group of Model: SHP2^MAC-KO^ group. EF, FS, LVAW in Model: SHP2^WT^ group compared to Sham: SHP2^WT^ group; d and LVAW; significantly reduced s, LVID; d and LVID; S is significantly elevated (Figure 1). It was suggested that myeloid-specific knockout of SHP2 can improve the cardiac function and reduce myocardial injury in mice with myocardial infarction.

### SHP2^MAC-KO^ could reduce myocardial infarct size in mice

**Figure 2:**
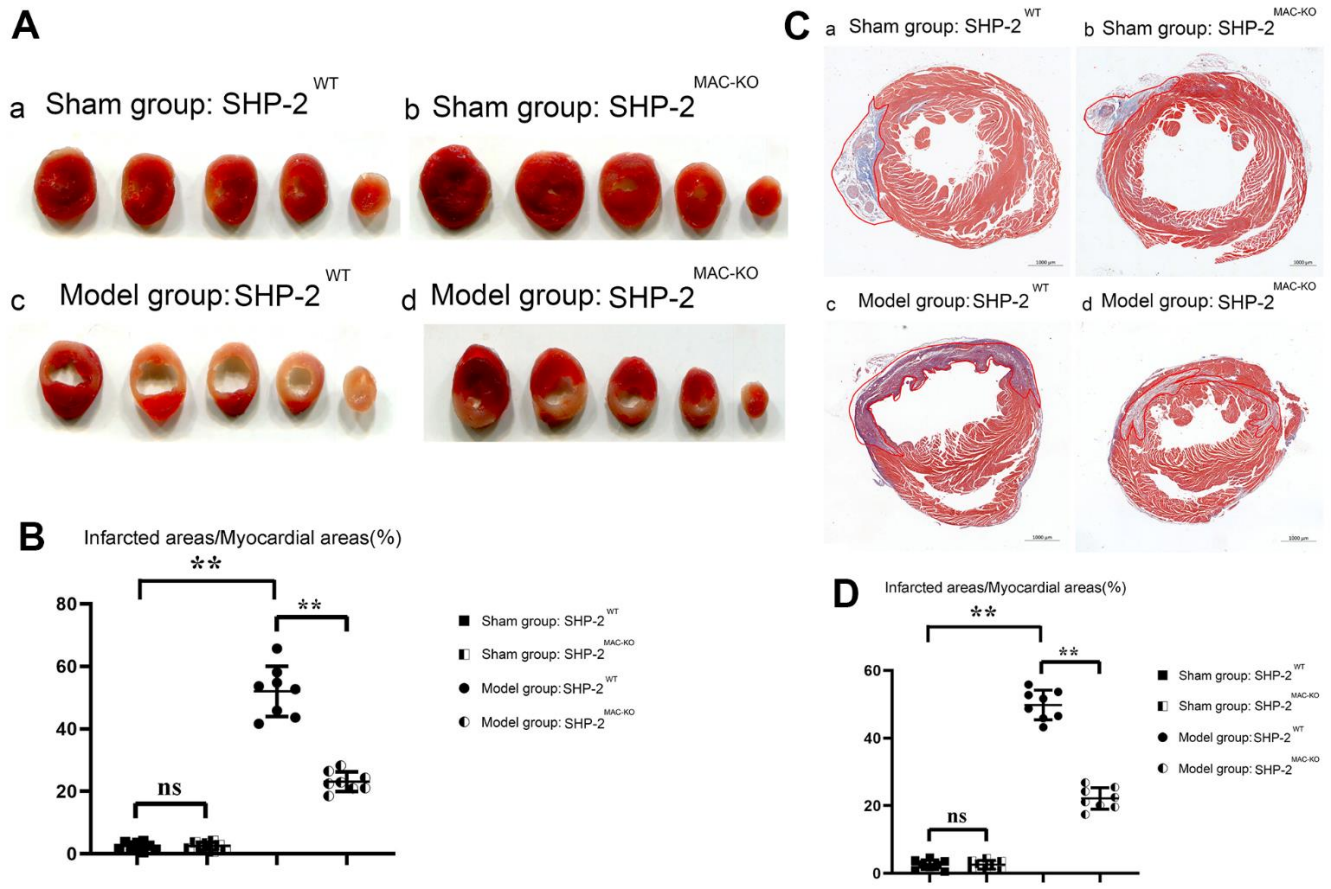
**SHP2^MAC-KO^ reduces myocardial infarct size in mice.** (**A**) TTC results; (**B**) percentage of infarct size in each group; (**C**) Plot of Masson Staining result; (**D**) Myocardial infarction area statistics. **P<0.01, ns P>0.05.

The results of TTC and Masson Staining showed that compared with the group of sham: SHP2^WT^, the size of myocardial infarction of mice in sham: SHP2^MAC-KO^ group was essentially unchanged. However, compared with the Model: SHP2^WT^ group, the infarct size of mice in Model: SHP2^MAC-KO^ group reduced significantly. Compared with the Sham: SHP2^WT^ group, the proportion of infarcts in the Model: SHP2^WT^ group was significantly higher. It was illustrated that the myocardial infarct size reduced significantly after myeloid-specific knockout of SHP2, and the myocardial cells were intact and myocardial morphology was normal in the sham: SHP2^WT^ and sham: SHP2^MAC-KO^ groups. In the Model: SHP2^WT^ group, myocardial cells were swollen, the nuclei were necrotic, and the inflammatory response and cell lysis were severe. However, in the Model: SHP2^WT^ group, the necrosis and swelling of cardiomyocytes was mild (Figure 2). These results indicated that myeloid-specific knockout of SHP2 may attenuate myocardial infarction injury in mice.

### SHP2^MAC-KO^ could reduce the number of apoptosis and inflammatory cell infiltration in mouse myocardial infarction

**Figure 3:**
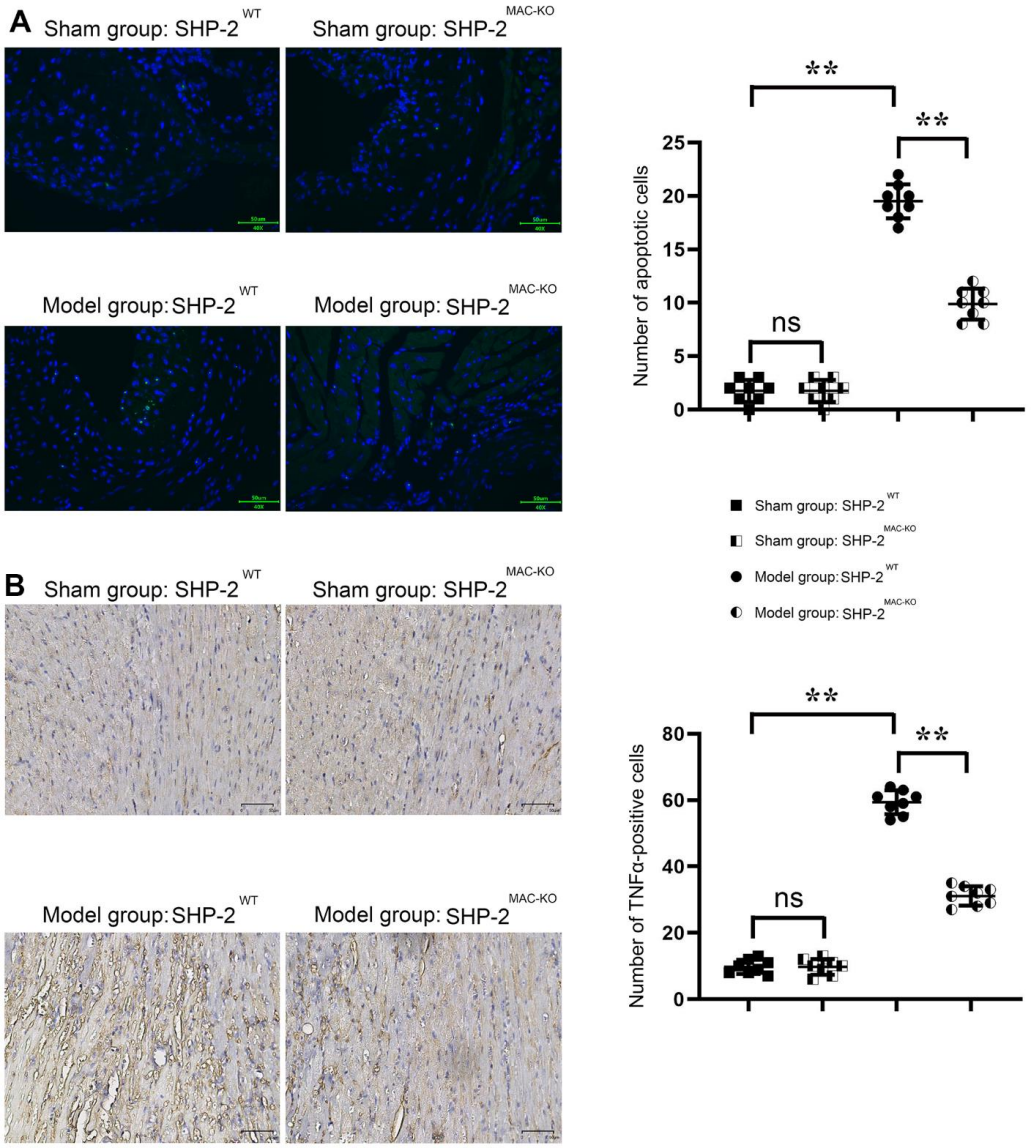
**SHP2^MAC-KO^ reduces the number of apoptosis and inflammatory cell infiltration in mouse myocardial infarction.** (**A**) Plots of TUNEL staining results and statistics of the number of apoptotic cells; (**B**) immunohistochemical staining results and relative fluorescence intensity data of TNFα. **P<0.01, ns P>0.05.

TUNEL staining showed that the number of apoptosis in the sham: SHP2^MAC-KO^ group was low and there was no significant difference compared with the sham: SHP2^WT^ group. Compared with the Model: SHP2^WT^ group, the number of apoptosis in the Model: SHP2^MAC-KO^ group was significantly reduced. The number of apoptosis in the Model: SHP2^WT^ group was significantly higher compared with the sham: SHP2^WT^ group. The results of immunohistochemical staining showed that there was no significant difference in the relative fluorescence intensity of TNFα in the sham: SHP2^MAC-KO^ group and the sham:SHP2^WT^ group, and the relative fluorescence intensity of TNFα was significantly reduced in the Model: SHP2^MAC-KO^ group compared with the Model: SHP2^WT^ group. The relative fluorescence intensity of TNFα in Model: SHP2^WT^ group was significantly higher than that of the sham: SHP2^WT^ group (Figure 3). Studies have shown that myeloid-specific knockout of SHP2 can reduce the number of apoptosis and infiltration of inflammatory cells in myocardial infarction.

### SHP2^MAC-KO^ can mediate PI3K/PLCγ signaling pathway to affect the expression of inflammatory factors and the level of protein for phagocytic activity

**Figure 4:**
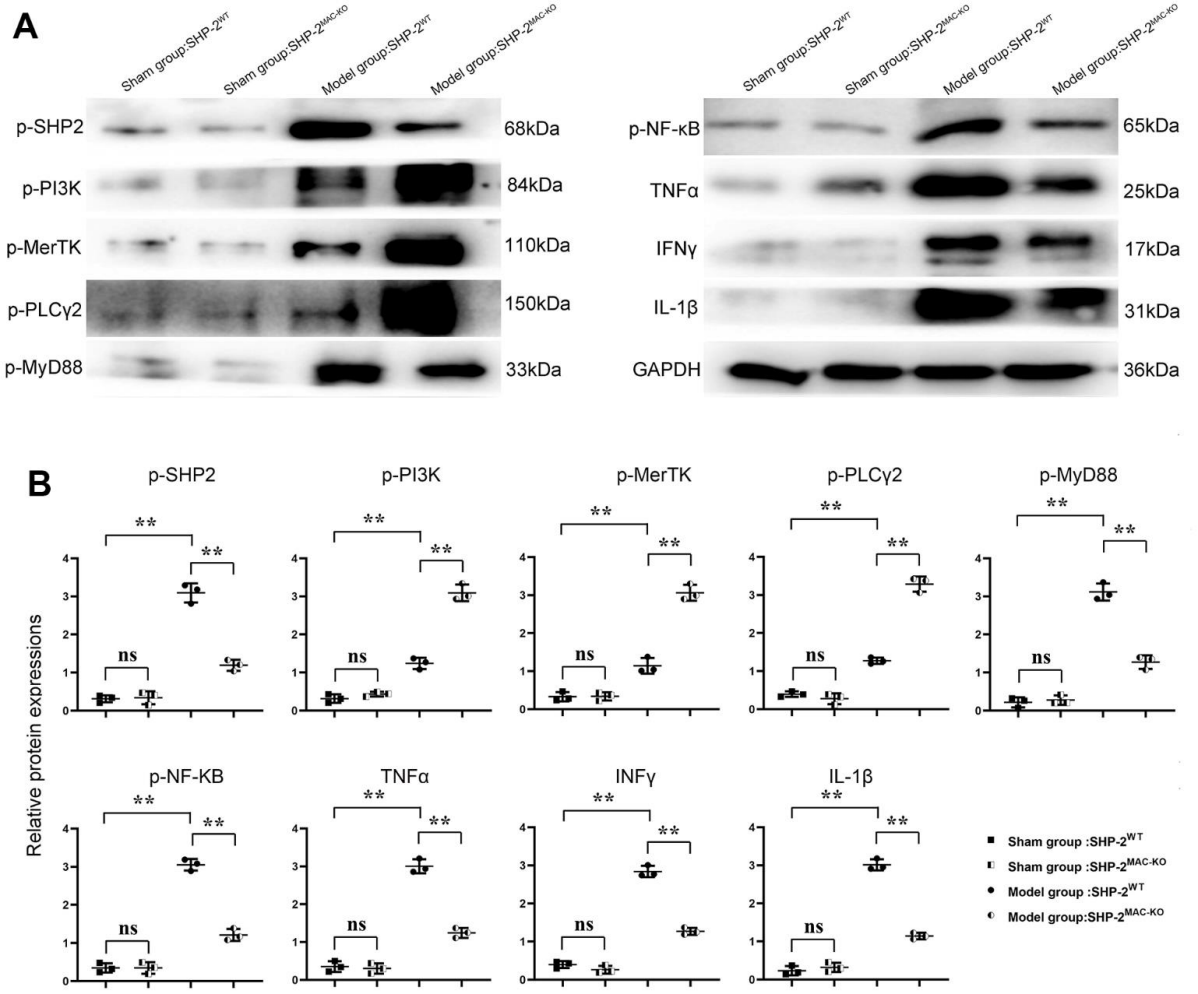
**SHP2^MAC-KO^ can affect the expression of PI3K/PLCγ signaling pathway, the expression of inflammatory factors and proteins related to phagocytic activity.** (**A**) Protein band diagram of p-SHP2, p-PI3K, p-MerTK, p-PLCγ2, p-MyD88, p-NF-κB, TNFα, IFNγ, IL-1β; (**B**) Relative protein expression of p-SHP2, p-PI3K, p-MerTK, p-PLCγ2, p-MyD88, p-NF-κB, TNFα, IFNγ, IL-1β. **P<0.01.

**Figure 5:**
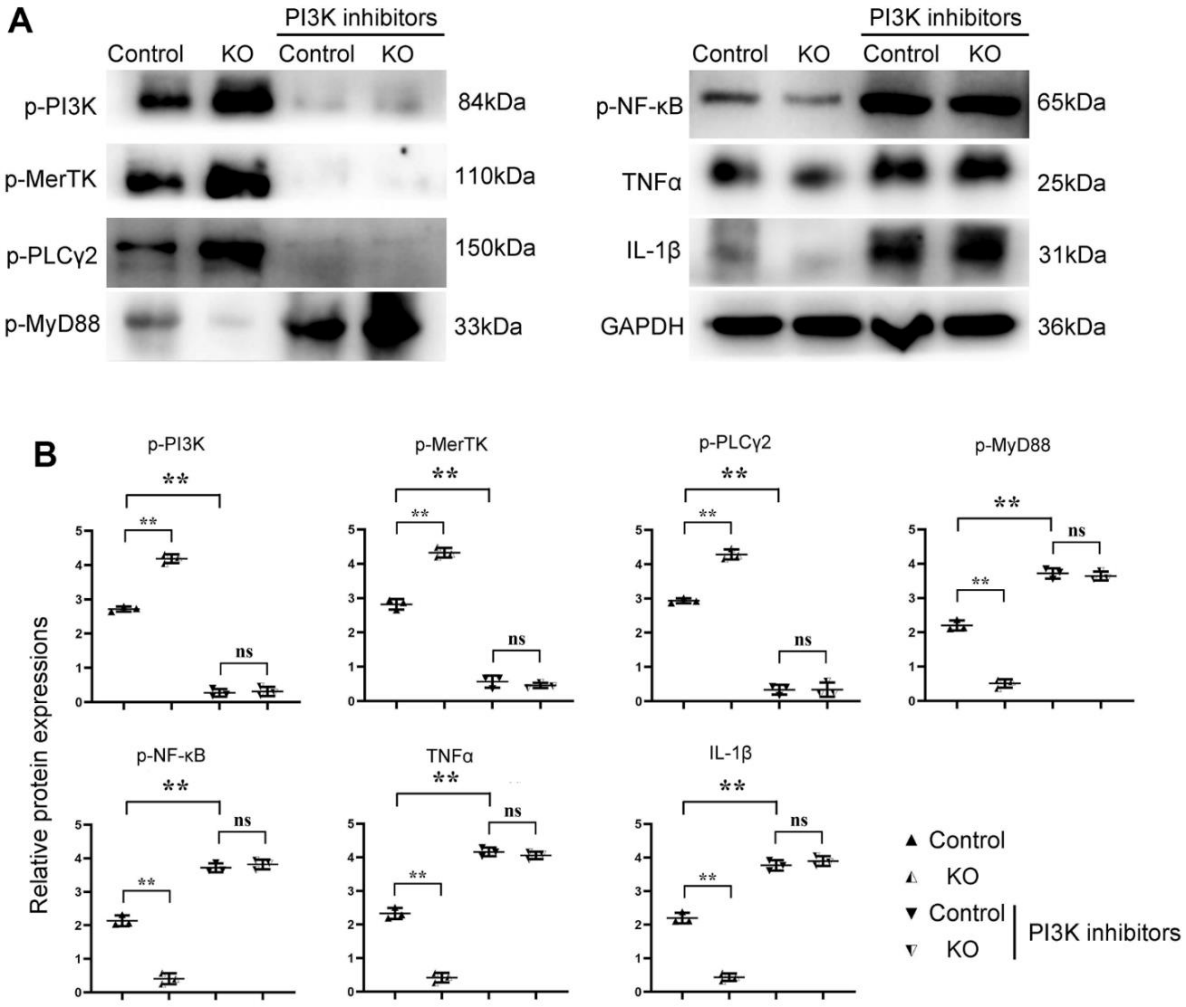
**SHP2^MAC-KO^ can mediate the PI3K/PLCγ signaling pathway to influence the expression of inflammatory factors and proteins related to phagocytic activity.** (**A**) Protein band diagram of p-PI3K, p-MerTK, p-PLC Γ2, p-MyD88, p-NF-κb, TNF α, IFN γ, il-1β; (**B**) Relative protein expression amounts of p-PI3K, p-MerTK, p-PLCγ2, p-MyD88, p-NF-κb, TNF α, IL-1β. **P<0.01, ns P>0.05.

The results of Western blotting showed that there was no significant difference in p-PI3K, p-MerTK, p-PLCγ2, p-MyD88, p-NF-κB, TNFα, IFNγ, and IL-1β in SHP2^MAC-KO^ in the sham group, and the expression of SHP2 was significantly reduced. Compared with Model: SHP2WT group, the expression of p-PI3K, p-MerTK and p-PLCγ2 significantly increased in SHP2^MAC-KO^ group, whereas the expression of p-SHP2, p-MyD88, p-NF-κB, TNFα, IFNγ, and IL-1β decreased significantly (Figure 4). These results manifested that myeloid-specific knockout of SHP2 mediated the PI3K/PLCγ signaling pathway to decrease the expression of inflammatory factors and promote the expression of protein related to phagocytic activity. The results of Western blotting *in vitro* showed that the expression of p-PI3K, p-MerTK and p-PLCγ2 in KO group was significantly higher than that in control group. The expression of p-MyD88, p-NF-κB, TNFα, IFNγ and IL-1β significantly decreased to the control group. However, there was no significant difference in the expression of these proteins between the control +PI3K inhibitor group and the KO+PI3K inhibitor group. Compared with the control group, the expression of p-PI3K, p-MerTK, p-PLCγ2 and p-MyD88, p-NF-κB, TNFα and IL-1β were significantly increased in the control+PI3K inhibitor group (Figure 5). It was verified that the mediation of PI3K/PLCγ signaling pathway by myeloid specific knockout SHP2 affected the expression of inflammatory factors and proteins related to phagocytic activity.

### SHP2^MAC-KO^ could mediate PI3K/PLCγ signaling pathway to enhance phagocytic activity of macrophages

**Figure 6:**
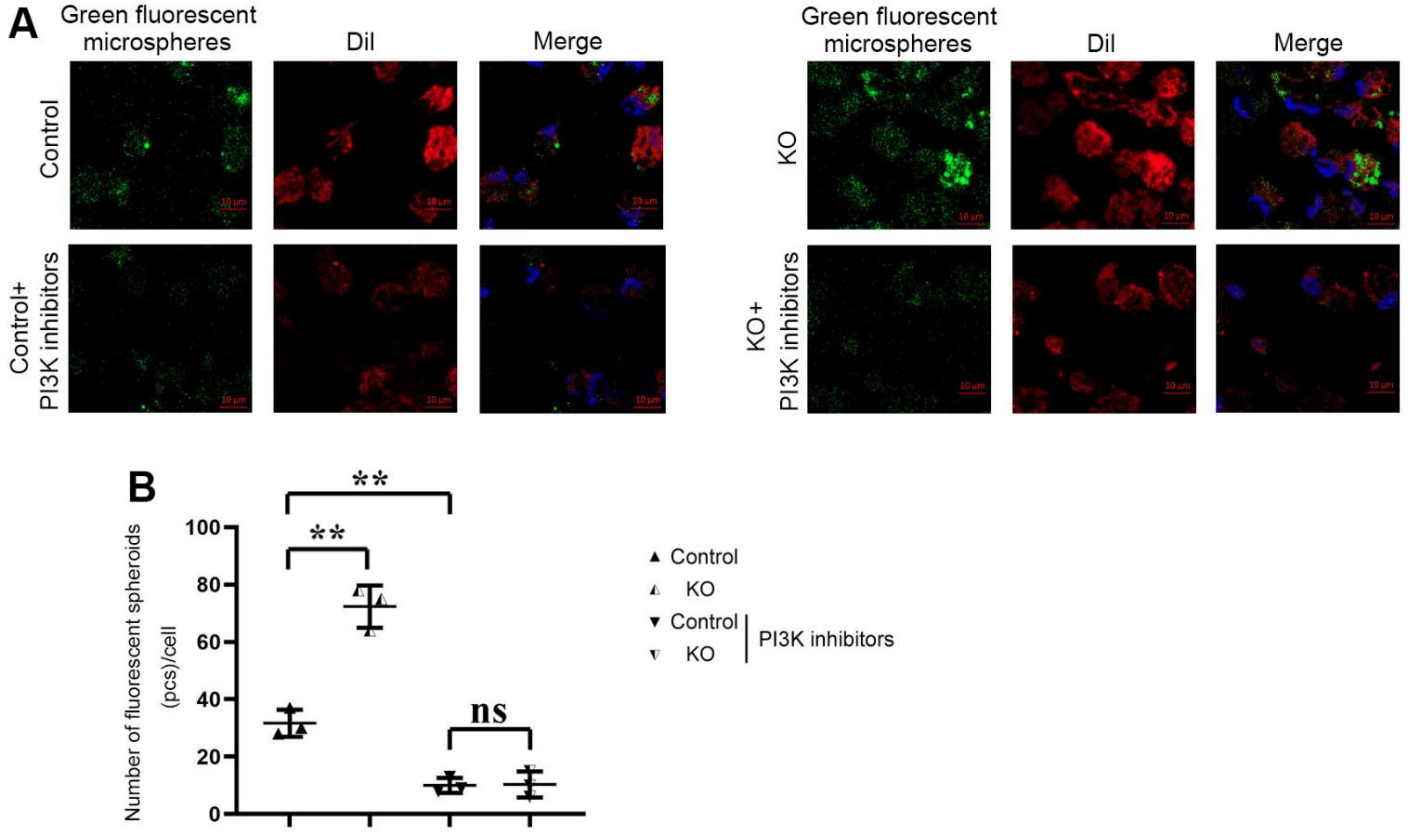
**Phagocytosis of macrophages detected by phagocytosis assay with fluorescent microspheres.** (**A**) Results of Phagocytosis Assay with Fluorescent Microspheres; (**B**) Statistics of Phagocytosis Assay with Fluorescent Microspheres. ** P<0.01, ns P>0.05.

The phagocytosis of macrophages in each group was detected by Phagocytosis Assay with Fluorescent Microspheres. DiI, namely DiIC18(3) with the full name 1,1’-dioctadecyl-3,3,3’,3’-tetramethylindocarbocyanine perchlorate, is one of the most commonly used cell membrane fluorescent probes, showing orange-red fluorescence. DiI is a lipophilic dye that can diffuse laterally after entering the cell membrane and gradually stain the entire cell membrane. The results showed that the number of green fluorescent microspheres phagocytosed by macrophages in KO group was significantly higher than that in control group. Compared with the control group, the number of fluorescent spheroids in the control+PI3K inhibitor group was significantly reduced. However, there was no big difference in the number of green fluorescent microspheres phagocytosed by macrophages between control+PI3K inhibitor group and KO+PI3K inhibitor group. It was demonstrated that myeloid-specific knockdown of SHP2 could mediate PI3K/PLCγ signaling pathway to enhance phagocytic activity of macrophages (Figure 6).

### SHP2^MAC-KO^ could mediate PI3K/PLCγ signaling pathway to attenuate the expression of inflammatory factors in macrophages

**Figure 7:**
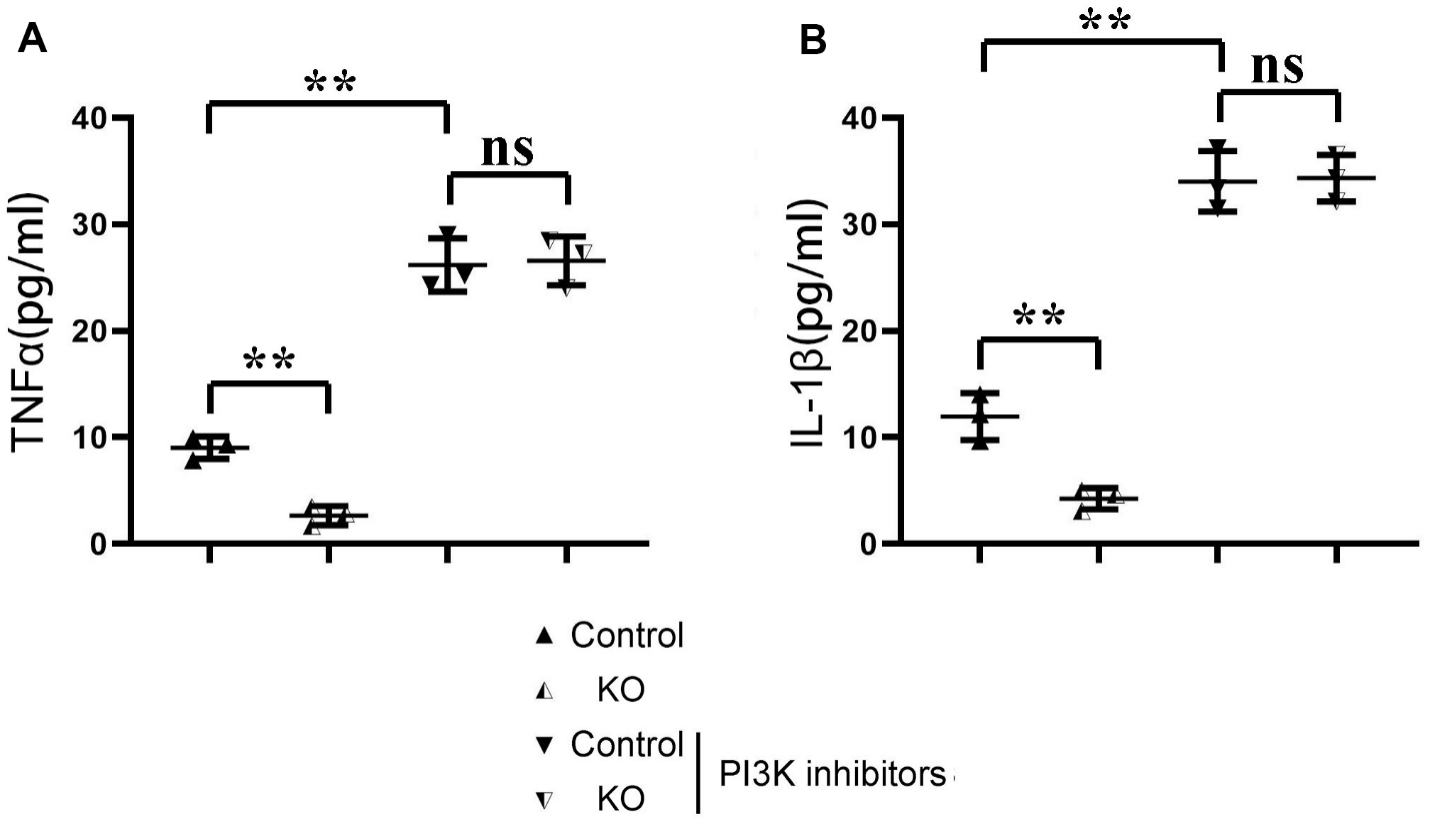
**ELISA detection of the content of inflammatory factors in macrophages in each group.** (**A**) Statistics of the TNF α; (**B**) Statistics of IL-1 β **P<0.01, ns P>0.05.

**Figure 8:**
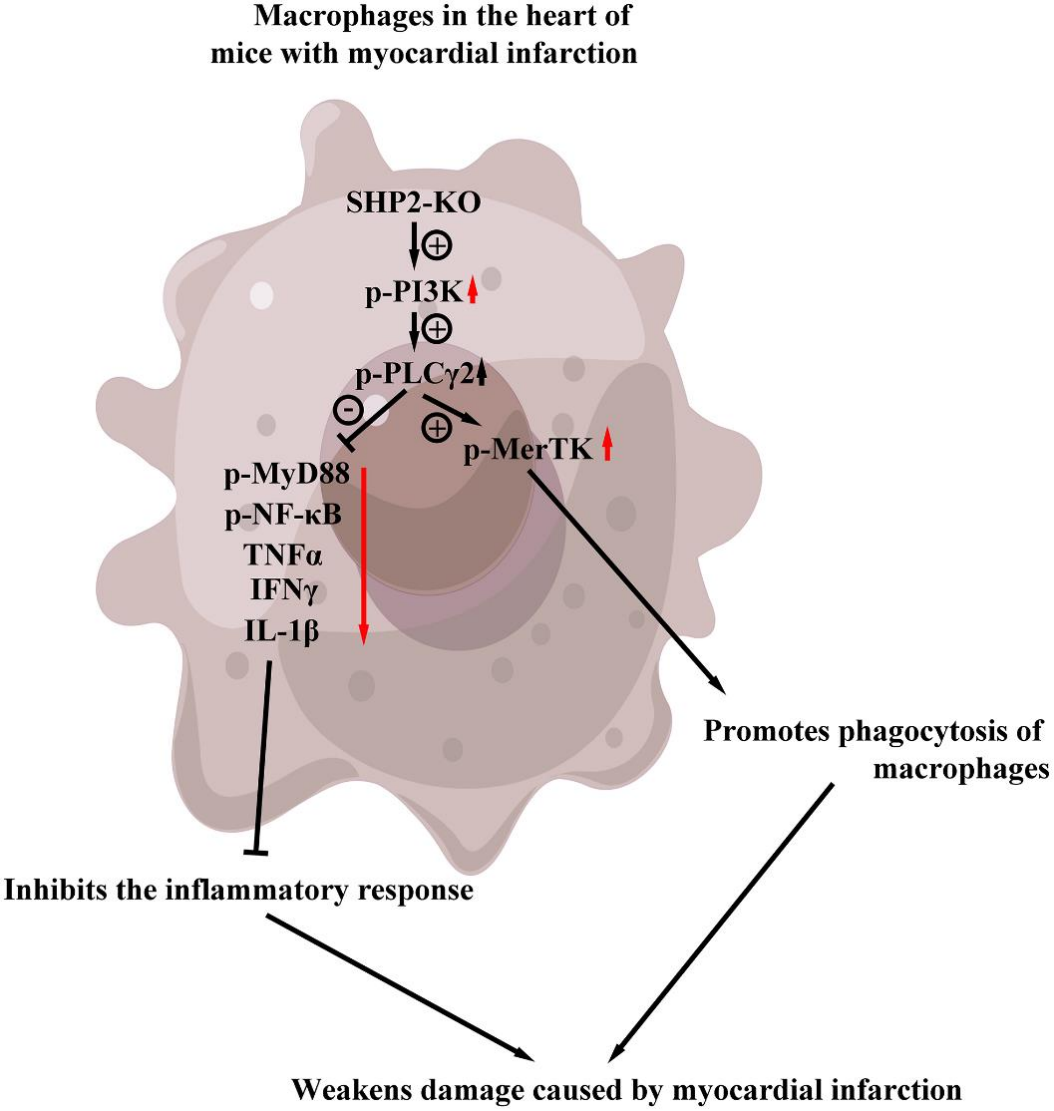
Myeloid-specific knockout SHP2 mediates the PI3K/PLCγ signaling pathway, affects the expression of inflammatory factors and the level of phagocytic activity, thereby promoting the phagocytic activity of macrophages and reducing the content of inflammatory factors, thereby protecting early myocardial infarction injury.

The results of ELISA showed that the contents of TNF-α and il-1β in KO group were significantly lower than those in the control group, but there was no significant difference in the contents of TNF-α and il-1β between the control+PI3K inhibitor group and KO+PI3K inhibitor group. It was demonstrated that myeloid-specific knockout of SHP2 could mediate the PI3K/PLCγ signaling pathway to inhibit the expression of inflammatory factors in macrophages (Figure 7). The above results show that myeloid-specific knockout SHP2 mediates the PI3K/PLCγ signaling pathway, affects the expression of inflammatory factors and the level of phagocytic activity, thereby promoting the phagocytic activity of macrophages and reducing the content of inflammatory factors, thereby protecting early myocardial infarction injury (Figure 8).

## DISCUSSION

Myocardial infarction is a clinically common cardiovascular disease brought by loss of blood supply to the myocardium attached to the branch coronary artery due to acute coronary syndrome and persistent hypoxia-ischaemia [20, 21]. Moreover, the heart failure (HF) after myocardial infarction remains a major problem in clinical practice. Failure to reduce inflammation after myocardial injury and unsuccessful left ventricular remodeling underlie the pathogenesis of heart failure [22]. As a result, it is very important to explore signaling pathways as new therapeutic targets for the development of myocardial infarction therapy.

SHP2 is an allosteric inhibitor consisting of two tandem-arranged SH2 domains (n-SH2 and c-SH2) in the N-terminal region of SHP2, one protein tyrosine phosphatase (PTP) catalytic domain, and a C-terminal domain [23]. In the basal state, the NH(2)-terminal SH2 domain of SHP-2 interacts with the PTP domain, resulting in autoinhibition of PTP activity [24]. NS and JMML, found at the interface of the N-SH2 and PTP domains, are caused by gain-of-function (GOF) mutations of SHP2, which abrogates the autoinhibition between the two domains. Loss-of-function (LOF) mutations in the PTP domain results in impaired PTP activity [25]. Interestingly, previous studies have shown that both GOF mutations and LOF mutations have an increased tendency to promote the open conformation of SHP2 [26]. However, the link between SHP2 and the molecular mechanisms underlying relevant human diseases has not been fully understood.

The receptor tyrosine kinase (RTK) MerTk (also known as c-Mer and Tyro12) translocates to the macrophage membrane and cytoskeletal fractions in a PS-R-dependent manner to induce phagocytosis of macrophages. Immunoprecipitation displays the association of PLC gamma2 with MerTK, and after the exposure to apoptotic cells, an intracellular signaling protein of PLCγ2 is recruited by wild-type MerTK [27]. Moreover, PLCγ2 has previously been shown to play an important role in (PLCγ2) interpretation of LPS-induced IP3 production and subsequent calcium release, leading to the activation of IRF3 [28]. Because active PLCγ hydrolyzes PIP2 and TIRAP anchors to PIP2 on the plasma membrane, PLCγ can prevent the recruitment of TIRAP to TLR4, and can form a complex with TLR4 in LPS-induced macrophages [29]. Therefore, it may be inferred that PLCγ2 can inhibit the expression of inflammatory factors in MyD88-NF-κB signaling pathway.

In this study, the ultrasound detection assessed whether myeloid-specific knockout of SHP2 has improved cardiac function in mice with myocardial infarction. The results showed that myeloid-specific knockout of SHP2 could increase ejection fraction (EF), shortening fraction (FS), diastolic left anterior wall thickness (LVAW; d) and systolic left anterior wall thickness (LVAW; s), and decrease left ventricular diastolic diameter (LVID; d) and left ventricular systolic diameter (LVID; s). It was suggested that myeloid-specific knockout of SHP2 can improve cardiac function in mice with myocardial infarction.

Nuclear necrosis, cell lysis, cell swelling, and severe inflammation are all possible effects of myocardial infarction. Through TTC and Masson Staining, it was found that myeloid-specific knockout of SHP2 could reduce the infarct size in myocardial tissue of mice and significantly reduce cell swelling and cell lysis. TUNEL staining and immunofluorescence staining results showed that myeloid-specific knockout of SHP2 could reduce the number of apoptosis and inflammatory cell infiltration in myocardial infarction. Through Western blotting, it was found that myeloid-specific knockout of SHP2 increased the expression of p-PI3K, p-MerTK and p-PLCγ2 and decreased the expression of p-MyD88, p-NF-κB, TNFα, IFNγ, IL-1β. The macrophages were treated with the PI3K inhibitor 740Y-P. Through phagocytosis assay with fluorescent microspheres, ELISA and Western blotting, the phagocytic activity of macrophages, the content of inflammatory factors, and the proteins associated with the PI3K/PLCγ signaling pathway in each group were examined. These results indicated that myeloid-specific knockout of SHP2 could mediate the PI3K/PLCγ signaling pathway to influence the expression of inflammatory factors and the level of protein for phagocytic activity, which can in turn promote phagocytic activity of macrophages and reduce the content of inflammatory factors.

In conclusion, through a series of *in vivo* and *in vitro* experiments, it was found that myeloid-specific knockout of SHP2 regulates PI3K/PLCγ signaling pathway to protect against early myocardial infarction injury.
